# Nanostructured Lipid Carrier Gel Formulation of Recombinant Human Thrombomodulin Improve Diabetic Wound Healing by Topical Administration

**DOI:** 10.3390/pharmaceutics13091386

**Published:** 2021-09-02

**Authors:** Yuan-Shuo Hsueh, Yan-Jye Shyong, Hsiu-Ching Yu, Shu-Jhen Jheng, Shang-Wen Lin, Hua-Lin Wu, Jui-Chen Tsai

**Affiliations:** 1 Department of Medical Science Industries, College of Health Sciences, Chang Jung Christian University, Tainan 711, Taiwan; yshsueh@mail.cjcu.edu.tw; 2 College of Bioscience and Biotechnology, National Cheng Kung University, Tainan 701, Taiwan; 3 International Center for Wound Repair and Regeneration, National Cheng Kung University, Tainan 701, Taiwan; 4 Institute of Clinical Pharmacy and Pharmaceutical Sciences, College of Medicine, National Cheng Kung University, Tainan 701, Taiwan; bear901704@gs.ncku.edu.tw (Y.-J.S.); shuhsiulovely@gmail.com (H.-C.Y.); kojiba419@gmail.com (S.-J.J.); shangwen19920125@gmail.com (S.-W.L.); 5 Department of Biochemistry and Molecular Biology, National Cheng Kung University, Tainan 701, Taiwan; halnwu@mail.ncku.edu.tw; 6 Blue Blood Cooperation, Taipei 110, Taiwan

**Keywords:** nanostructured lipid carrier, protein drug delivery, sustained release, chronic wound healing, angiogenesis factor, carbopol gel, thrombomodulin

## Abstract

Recombinant human thrombomodulin (rhTM), an angiogenesis factor, has been demonstrated to stimulate cell proliferation, keratinocyte migration and wound healing. The objective of this study was to develop nanostructured lipid carrier (NLC) formulations encapsulating rhTM for promoting chronic wound healing. RhTM-loaded NLCs were prepared and characterized. Encapsulation efficiency was more than 92%. The rate of rhTM release from different NLC formulations was influenced by their lipid compositions and was sustained for more than 72 h. Studies on diabetic mouse wound model suggested that rhTM-NLC 1.2 µg accelerated wound healing and was similar to recombinant human epidermal growth factor-NLC (rhEGF-NLC) 20 µg. By incorporating 0.085% carbopol (a highly crosslinked polyacrylic acid polymer) into rhTM NLC, the NLC-gel presented similar particle characteristics, and demonstrated physical stability, sustained release property and stability within 12 weeks. Both rhTM NLC and rhTM NLC-gel improved wound healing of diabetic mice and cell migration of human epidermal keratinocyte cell line (HaCaT) significantly. In comparison with rhTM solution, plasma concentrations of rhTM post applications of NLC and NLC-gel formulations were lower and more sustained in 24 h. The developed rhTM NLC and rhTM NLC-gel formulations are easy to prepare, stable and convenient to apply to the wound with reduced systemic exposure, which may warrant potential delivery systems for the care of chronic wound patients.

## 1. Introduction

The wound healing process is a physiological dynamic interaction which involves several types of cells, tissues or proteins, such as parenchymal cells, blood cells, extracellular matrix, cytokines networks and growth factors, aiming at repairing the damaged cutaneous tissues [1,2,3]. Wound healing events can be divided into four phases: hemostasis, inflammatory, proliferative and remodeling phases [1,2,3,4,5,6]. The hemostasis phase leads to vasoconstriction and platelets aggregation at the site of injury to prevent exsanguination. The inflammatory phase is represented by a complex series of molecular signals that facilitates neutrophil and monocyte infiltration in order to prevent unnecessary tissue damage and eliminate pathogenic organisms and foreign debris. The main events of the proliferative phase are epithelialization, angiogenesis, granulation tissue formation and collagen deposition. The final tissue remodeling phase is characterized by the transition from granulation tissue to scar formation. However, wound healing is impaired in chronic wounds, such as seen in diabetes patients. Chronic wounds fail to heal due to alteration of one or more biological processes [6]. For instance, inappropriately amplified and prolonged high levels of proinflammatory cytokines in the wound lead to high-level expression of matrix metalloproteinases. These subsequently destroy the following wound cascade such as signaling or expression of growth factors, receptors and matrix proteins for wound healing.

Various growth factors, cytokines, proteins and cells influence the tissue repair process. Epidermal growth factor (EGF) and its receptor (EGFR) play an essential role in wound healing, and have been successfully used in the process of wound healing. Through stimulating the growth and differentiation of keratinocytes as well as the proliferation and migration of fibroblasts and vascular endothelial cells, EGF promotes the formation of granulation tissue and re-epithelialization [2,4,7,8]. Human recombinant thrombomodulin (rhTM) is a protein with EGF-like domain. It has been reported to stimulate cell proliferation, migration, and capillary-like tube formation in human umbilical vein endothelial cells [9]. Local administration of rhTM enhances fibroblast growth factor receptor 1 activation and increases vascular density in the rat hindlimb ischemia model [10]. These findings indicate that rhTM is a potent angiogenic factor in vivo. Moreover, the epidermis-specific TM knockout mice display delayed wound healing with less neovascularization and reduced epithelial proliferation when compared to control mice [11]. The administration of rhTM improves wound repair in the TM knockout skin, in high-glucose cultured keratinocyte cells and diabetic mice [11,12]. Importantly, rhTM was demonstrated as a more stable and potent protein drug than EGF for wound healing [13]. Therefore, rhTM is a promising therapeutic agent for treatment of chronic wounds.

Lipid-based drug delivery systems have been widely used and attracted strong interest in the formulations administered via various delivery routes. It has the advantages of increasing drug solubility, targeting, reducing side effects and the ability of controlled release [14]. Among those systems, nanostructured lipid carriers (NLC), a second generation lipid nanoparticle, provide higher drug loading capacity and drug release efficiency than solid lipid nanoparticles (SLN) [15,16,17]. It is a mixture of lipid–oil matrix, which is easy to prepare forming a nano-sized sphere structure. It was reported that rhEGF loaded in NLC was capable of improving wound healing in genetic knockout diabetic mice when compared with the free form of rhEGF [16]. Similar results were observed in the porcine model [18,19]. Based on these studies, NLC is rationalized as a safe and suitable carrier for rhTM cutaneous delivery.

Synthetic polymer gels are common formulations which combine with drugs or drugs carriers to increase viscosity and residence time during cutaneous treatment [20,21]. They are usually transparent or semi-transparent, and do not have the oily and uncomfortable feel as most oil-based ointments or creams have. Some polymer gels may increase cell proliferation and migration, which is beneficial for wounding healing [22,23]. Previous studies have used gel mixed with lipid nanoparticles to prevent skin irritation, and to deliver drugs into the deeper layers of skin. It can increase the dispersion and stability of lipid nanoparticles, making it easier to administrate on skin for psoriasis treatment [24]. These indicated that it can be advantageous by combining gel with NLC for the treatment of chronic wounds.

The purpose of this study was to develop a NLC-gel formulation as cutaneous delivery system of rhTM for chronic wound healing with sustained-release characteristics. At first, rhTM was loaded into NLC to provide a consistent sustained-release up to 72 h. Carbopol is a highly crosslinked polyacrylic acid polymer, which is commonly used as viscosity-enhancing agent. In clinical experience, the loss of rhTM NLC solution during would healing may become an issue. This is because rhTM NLC is liquid in form, and does not have the viscosity to prevent loss due to body motion. It could also dry out easily due to vaporization. Therefore in this study, rhTM NLC was then incorporated into carbopol gel forming rhTM NLC-gel, to prevent easy removal of the formulation after administration on skin; also providing a moist environment beneficial for wounding healing. We characterized the formulation properties by evaluating particle size and zeta potential through dynamic light scattering (DLS). The morphology of the formulation was visualized by transmission electron microscopy (TEM). In vitro drug release of rhTM in NLC and NLC-gel was performed using centrifuge technique and diffusion cell, respectively. Their efficacy on diabetic mice by wound closure percentage and skin histology. Effect on migration of HaCaT cells was performed to delineate possible contribution from different formulation components. Plasma concentrations of rhTM after applications of NLC formulations were determined.

## 2. Materials and Methods

### 2.1. Materials

Precirol ATO5 (glyceryl palmitostearate) was obtained from Gattefosse (Saint-Priest, France) and Miglyol 812 was received from Sasol (Hamburg, Germany). Carbopol 940 was purchased from Acros (Morris County, NJ, USA). Triethanolamine (TEA) was purchased from Merck (Darmstadt, Germany). RhTM protein and anti-rhTM antibody were provided by Blue Blood Biotech Corporation (Taipei, Taiwan). H&E stain, Poloxamer 188 (10% solution), bovine serum albumin (BSA) and phosphate buffered saline (PBS) were purchased from Sigma-Aldrich (St. Louis, MO, USA). Streptozotocin (STZ) was obtained from Cayman Chemical (Ann Arbor, MI, USA). DMEM and fetal bovine serum (FBS) were purchased from GE Healthcare (Pittsburgh, PA, USA). Formaldehyde solution was from Avantor (Center Valley, PA, USA), and penicillin-streptomycin was from Gibco (Waltham, MA, USA).

### 2.2. Preparation and Characterization of RhTM NLC and RhEGF NLC

NLCs containing 2% total lipids and 0.67% poloxamer 188 as surfactant were prepared by hot homogenization method. Briefly, various ratios of solid lipids and oil were mixed and melted under 75 °C as oil phase. RhTM or rhEGF was dissolved in aqueous phase containing poloxamer 188 under 37 °C. Then the oil phase was mixed with aqueous phase and emulsified using a probe sonicator (Misonix S3000) for 30 s (output = 50 W), forming rhTM NLC or rhEGF NLC. The formulation was cooled to room temperature thereafter, and its weight was adjusted with water. Final concentrations were rhTM 24 μg/g, rhEGF 24 μg/g and 400 μg/g in the NLC formulations, respectively. 

For characterization of various rhTM NLC formulations, 300 µL of sample was diluted 10 times with water and particle size was analyzed with dynamic light scattering (DLS, Nanoplus zeta/nano particle analyzer, Particulate System). Laser doppler micro electrophoresis was used for zeta potential measurement. Morphology of rhTM NLC was observed by transmission electron microscope (TEM) under the assistance of Center for Micro/Nano Science and Technology, National Cheng Kung University. One to otw omicroliters of sample was sealed into liquid cell for TEM observation.

### 2.3. Preparation of RhTM NLC Carbopol Gel

An amount of 0.017 g of Carbopol 940 powder and 3 g of 0.04% of TEA were added into 7 g of water and stirred for 2 days until well distributed. Then the gel was aged for 1 day for bubble removal. RhTM NLC-gel was prepared by mixing 2-fold concentration of rhTM NLC with carbopol gel in a 1:1 ratio for 30 min, and stored at 4 °C for further usage. Particle size, zeta potential and morphology of rhTM NLC-gel were analyzed by DLS and TEM as described above for rhTM NLC. Viscosity of carbopol gel was measured by Cannon-Fenske viscometer (no. 200). The prepared NLC-gel formulations contained 24 and 60 μg/g of rhTM. 

### 2.4. Loading Efficiency and In Vitro Drug Release Profile of RhTM NLC and RhTM NLC-Gel

Drug loading efficiency of rhTM NLC was estimated indirectly by measuring the free rhTM (non-encapsulated) removed through the filtration/centrifugation technique modified from Gainza et al. [18]. Briefly, 20 µL of rhTM NLC was added with 2 mL 0.005% BSA in pH 7.4 PBS solution into a 100 kDa centrifugal filter tube (Sartorius, Gottingen, Germany), and centrifuged for 40 min under 2500 rpm. Solution from the lower compartment of the tube was collected for ELISA analysis, and drug loading efficiency was determined using the formula below:

(1) $$\mathrm{Drug}\;\mathrm{loading}\;\mathrm{efficiency}\;(\%)=\frac{\mathrm{Nominal}\;\mathrm{drug}\;\mathrm{amount}\;\mathrm{in}\;\mathrm{formulation}\;-\;\mathrm{Drug}\;\mathrm{amount}\;\mathrm{in}\;\mathrm{solution}}{\mathrm{Nominal}\;\mathrm{drug}\;\mathrm{amount}\;\mathrm{in}\;\mathrm{formulation}}\;\times \;100\%$$

To determine the release of rhTM from NLC formulation, 20 µL of rhTM NLC was added with 2 mL 0.005% BSA/PBS solution into 100 kDa centrifugal filter tube and incubated at 37 °C, 50 rpm in a hybridization oven. At selected intervals for 3 days, solution from the lower compartment of the tube was removed by centrifugation for 40 min under 2500 rpm and replaced by same quantity of 0.005% BSA/PBS. 

Drug release profile of rhTM NLC-gel was determined using flow-through diffusion cells (Laboratory Glass Apparatus, Berkeley, CA, USA) with 1 cm^2^ of diffusion area and 3.6 mL of receiver volume, stirred at 700 rpm and maintained at constant temperature by a 37 °C circulating water bath. In brief, dialysis membrane was locked between donor and receptor chamber. The receptor chamber was filled with 0.005% BSA/PBS, and 300 µL of rhTM NLC-gel sample was added into donor chamber. At 3, 6, 12 and 24 h by injecting 0.5 mL of 0.005% BSA/PBS into receptor chamber, samples were collected from the outlet of the chamber. 

A sandwich ELISA assay was used to measure rhTM concentration. In brief, a capture antibody (poly-IgY) 100 µL/well was coated on to 96-well plate. After blocking, 100 µL/well of sample were added and incubated at 37 °C for 2 h. Primary antibody was added for 2 h, followed by secondary antibody for 1 h. After multiple washing with PBST, staining agent was added for 30 min, and rhTM concentration was analyzed by UV-vis at wavelength of 450 nm. 

### 2.5. Wound Healing Experiments

Male C57BL/6J mice (8–12 weeks old, National Laboratory Animal Center, Taiwan) were housed under conditions of controlled humidity (40%) and temperature (22 ± 2 °C) with 12-h light-dark cycles. All procedures using animals were approved by the Institutional Animal Care and Use Committee (IACUC) of National Cheng Kung University (IACUCapproval numbers 107069 and 109128, approved on 29 December 2017 and 19 February 2020, respectively). Detailed protocol has been described previously [13]. Briefly, mice were allowed free access to food and water until 4–6 h prior to streptozotocin treatment. They were intraperitoneally (IP) injected with 50 mg/kg of streptozotocin for 5 consecutive days to induce diabetes. Blood glucose levels above 300 mg/dL were confirmed using a glucose meter (Bionime GM550) before further experiments. The hair of diabetic mice on the dorsal area was removed under anesthesia with Zoletil (10 mg/mL, 0.05–0.06 mL/10 g BW), and 0.1 mL prophylactic antibiotics of penicillin-streptomycin (10,000 U/mL, Gibco) was given. Then, a rubber O-ring (inner diameter of 12.8 mm, outer diameter of 17.1 mm, 2.4 mm thickness) was adhered on the dorsal midline with cyanoacrylate adhesive (Instant glue, 3M 6886). An 8-mm diameter round-shaped, full-thickness excisional wound was made within the O-ring. Dorsal sites were selected for usefulness to keep the animal from reaching and manipulating the wound [25]. After surgery, wound beds were rinsed with PBS, and applied with 50 μL/dose of different treatments. 

**Figure 1:**
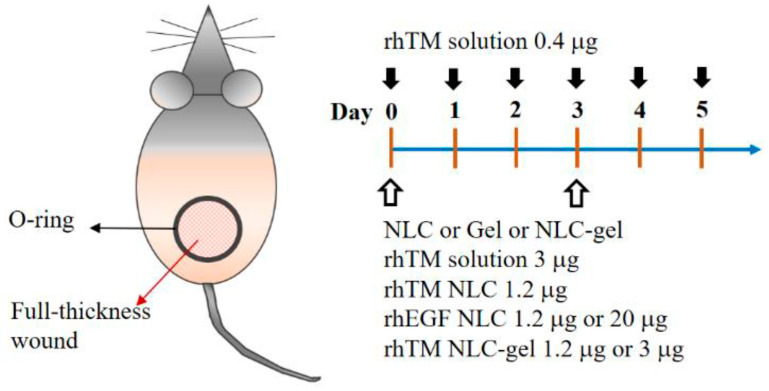
Topical treatment protocol. The black arrows denoted application days for rhTM solution 0.4 μg. The white arrows indicated application days for NLC, Gel, NLC-gel, rhTM solution 3 μg, rhTM NLC 1.2 μg, rhEGF NLC 1.2 μg, rhEGF NLC 20 μg, rhTM NLC-gel 1.2 μg and rhTM NLC-gel 3 μg, respectively. Each dose was applied in 50 μL solution or gel.

There were two sets of wound healing experiments. The first set was designed to compare between rhTM and rhEGF with and without NLC encapsulation. Animals were randomly divided into five groups: (i) NLC only, (ii) rhTM solution 0.4 µg, (iii and iv) rhEGF NLC 1.2 and 20 µg and (v) rhTM NLC 1.2 µg. RhTM solution 0.4 µg (group ii) was administrated daily to the wound up to 10 days as indicated with black arrows. The rest were administrated every 3 days indicated by white arrows (Figure 1). In the second set, NLC-gel was added into the study for efficacy evaluation. Animals were randomly divided into six groups: (i) Gel, (ii) NLC-gel, (iii) rhTM solution 3 µg, (iv) rhTM NLC 1.2 µg, (v and vi) rhTM NLC-gel 1.2 and 3 µg, respectively. Tegaderm film was covered over the rubber O-ring and circumferentially around the trunk of the animal. All study groups were administrated every 3 days indicated by white arrows (Figure 1). Wound areas were photographed each day and analyzed using ImageJ 1.48 software (Nation Institutes of Health, Bethesda, MD, USA) by an experienced person and verified by another. The percentage of wound closure was calculated by the following equation.

(2) $$\mathrm{Wound}\;\mathrm{Closure}\;(\%)=\frac{\mathrm{Wound}\;\mathrm{Area}\;\left(\mathrm{Day}\;1\right)-\mathrm{Wound}\;\mathrm{Area}\;\left(\mathrm{Day}\;n\right)}{\mathrm{Wound}\;\mathrm{Area}\;\left(\mathrm{Day}\;1\right)}\times 100\%$$

### 2.6. Histology and Masson’s Trichrome Staining

Detailed protocol was described previously [13]. Briefly, wounded skins of sacrificed mice on day 10 were collected, fixed, and embedded in paraffin. The samples were stained with hematoxylin and eosin (H&E). Masson’s trichrome staining was performed following a protocol of staining kit (HT15; Sigma-Aldrich). The sections were incubated with Bouin’s solution, Weigert’s iron hematoxylin, Biebrich Scarlet-Acid Fuchsin, phosphotungstic/phosphomolybdic acid solution, aniline blue solution and 1% (*v/v*) acetic acid, for various time and rinsed, respectively. The sections were dehydrated, mounted and photographed using a TissueGnostics GmbH FACS-like Tissue Cytometry. 

### 2.7. Immunofluorescence Staining

The paraffin-embedded mouse skin wound tissues were de-paraffined and soaked in descending alcohol. Tissue sections were boiled in Tris-EDTA buffer (10 mM Tris base, 1 mM EDTA solution, 0.05% Tween 20, pH 9.0) for antigen retrieval. The sections were incubated with 1% BSA for blocking 1 h at room temperature, primary antibody (CD31, ab28364, Abcam) overnight at 4 °C, then secondary antibody labeled with fluorophores 1 h at room temperature. DAPI was used as a nuclear marker. Slides were mounted and photographed using a TissueGnostics GmbH FACS-like Tissue Cytometry.

### 2.8. In Vivo RhTM Plasma Concentration

Animals were randomly divided into five groups: (i) NLC-gel, (ii) rhTM solution 3 µg, (iii) rhTM NLC 1.2 µg and (iv and v) rhTM NLC-gel 1.2 and 3 µg. Fifty microliters of formulations were administered on to wounds of diabetes mice, and blood samples were collected through submandibular vein at 30 min or 4 h into EDTA-rinsed tubes. At 24 h after administration, mice were euthanized and blood samples were collected through heart. All blood samples were centrifuged at 3000× *g* for 10 min, and plasma samples were collected for ELISA analysis using the assay described above.

### 2.9. In Vitro Cell Migration Assay

The human keratinocyte cell line, HaCaT, was maintained in DMEM with 10% fetal bovine serum (Hyclone) at 37 °C in humidified 5% CO_2_/95% air incubator under normal oxygen conditions. 5 × 10^4^ cells in 70 μL medium were seeded into each well of Culture-Insert 2 Well (ibidi, Gräfelfing, Germany) for 24 h. Cells were then incubated with indicated treatments including Gel, NLC, NLC-gel, rhTM solution 3 μg, rhTM NLC 1.2 μg, rhTM NLC-gel 1.2 μg and rhTM NLC-gel 3 μg, at 3000 fold dilution in serum-free medium for 48 h, and were photographed using OPTIKA camera and Leica microscopy system. The ratio of wound recovery was quantitatively determined with ImageJ software (National Institutes of Health, Bethesda, MD, USA). 

### 2.10. Statistical Analysis

Results from in vitro studies, cellular experiments, and rhTM plasma concentration were expressed as means ± standard deviation (SD), and wound closure percentage were presented as means ± standard error of the mean (SEM). All of the computations were performed using SPSS 17.0 (SPSS, Inc., Chicago, IL, USA). General linear model (GLM) was used for analysis of multiple comparisons of wound healing effect between groups, and Bonferroni method was used for post hoc test. Cell migrations between treatments were compared through student’s t test. Differences were considered statistically significant at *p* < 0.05.

## 3. Results and Discussion

### 3.1. RhTM NLC Characteristics

Table S1 listed all the excipients tested for NLC preparation. ATO5 and Geleol were used as solid lipids, Miglyol and Captex were used as liquid oils. Ratio of lipid and oil tested ranged from 8:2 to 4:6. Zeta potential of all the formulations was negative resulting from ionization of fatty acids in the lipids [24], and ranged from −25 mV to −45 mV. Zeta potential of NLC formulated with ATO5 had higher absolute value than NLC formulated with Geleol, and NLC formulated with higher ratio of oils showed more negative potential in comparison with lipids (Table S1 and Figure S1). NLCs formulated with ratio of lipid to oil 5:5 (1:1) exhibited smallest and more consistent particle size around 200 nm and polydispersity index (PDI) of 0.2–0.3, and were selected for further studies.

Characteristics of rhTM NLCs (lipid:oil = 5:5) were shown in Table S2. After encapsulating rhTM into NLC, no significant difference was found in particle size, PDI and zeta potential. Encapsulation efficiency of all four formulations was greater than 92% (Table S3). All the four rhTM NLCs released drug steadily in vitro, with NLC of ATO5/Miglyol (AM) showing the highest release of 68% within 72 h (Figure S2), similar to the extent of release of rhEGF-NLC reported previously [18]. The AM formulation was then selected as the test formulation for further studies.

### 3.2. RhTM NLC Is More Potent on Wound Healing than RhEGF NLC

**Figure 2:**
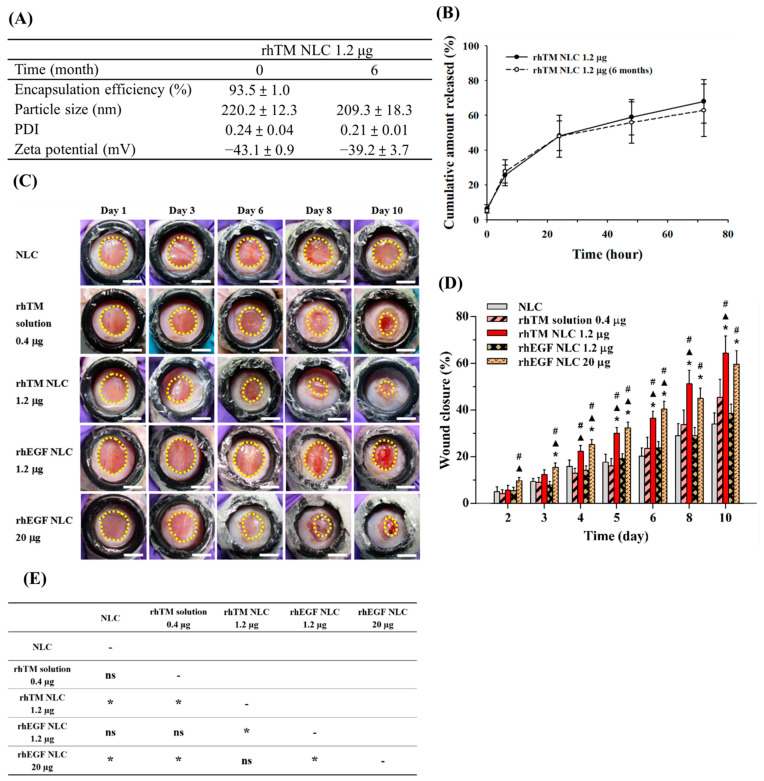
Characterization and wound healing effect of rhTM NLC 1.2 μg. (**A**) Physical characteristics of rhTM NLC 1.2 μg (*n* = 3). Data presented as mean ± SD. (**B**) In vitro drug release of freshly prepared rhTM NLC 1.2 μg and after storage at 4 °C for 6 months. Data presented as mean ± SD. (**C**) Representative wound area of streptozotocin-induced diabetic mice at Day 1 to 10 with different treatments. (*n* = 6–15 for each group; Scale bar = 5 mm). (**D**) Wound closure rate by quantifying wound area and analyzing with mathematical formula described in Methods. Data presented as mean ± SEM. The significance was analyzed using Student’s *t* test. *p* < 0.05 compared with NLC group (*), rhTM solution 0.4 µg group (▲), or rhEGF NLC 1.2 µg group (#). (**E**) Multiple comparison analysis of wound closure effect between different treatment groups based on general linear model. Data presented as *p* value. Bonferroni post hoc test was applied. (*: *p* < 0.05; ns: *p* > 0.05).

Physical characteristics of AM-based rhTM NLCs were shown in Figure 2A. Similar characteristics and in vitro drug profile of rhTM NLCs was found after 6 months of preparation, demonstrating a stable formulation with ability to protect rhTM degradation within 6 months (Figure 2B). Then, we verified the wound healing effect of rhTM NLC on streptozotocin-induced diabetic mice. Wound contraction is the main process during wound healing in rodent such as mice, and is quite different compared to human’s which mainly relies on re-epithelialization and granulation tissue formation [25,26,27]. To minimize wound contraction and to mimic the process of human wound healing, we have developed a skin wound healing model by using an O-ring to fix the skin around the wound opening. The model, which does not require delicate surgical sutures as in the splinted skin wound healing model, simplified the procedures. The percentage of wound contraction was evaluated on day 10, and ranged 16–32%, which was comparable with approximately 20% on day 11 reported for the splinted model [28]. This ring skin wound healing model made the mice a more feasible animal model for wound healing study [13]. The procedure of wound healing experiments is shown in Figure 1. Based on the results of in vitro studies, rhTM solution 0.4 μg, rhTM NLC 1.2 μg, rhEGF NLC 1.2 μg and rhEGF NLC 20 μg were chosen to evaluate their effect on wound healing in vivo (Figure 2C). Wound healing rates at different days were quantified and shown in Figure 2D. At Day 10, mice treated with rhTM-NLC 1.2 μg (64.4 ± 7.3%) had significantly better wound healing than those treated with NLC control (34.1 ± 4.7%), rhTM 0.4 μg solution (45.4 ± 7.8%) or rhEGF NLC 1.2 μg (38.6 ± 3.9%). Moreover, rhTM-NLC 1.2 μg and rhEGF 20 μg (59.7 ± 5.9%) showed no difference in wound healing. Furthermore, rhTM-NLC 1.2 μg stored at 4 °C after one year had a similar effect on wound healing in comparison with the freshly prepared formulation (Figure S3). Figure 2E summarized the significances from multiple comparison analysis of wound healing effects between all study groups. The results were consistent with comparisons based on Day 10 data.

**Figure 3:**
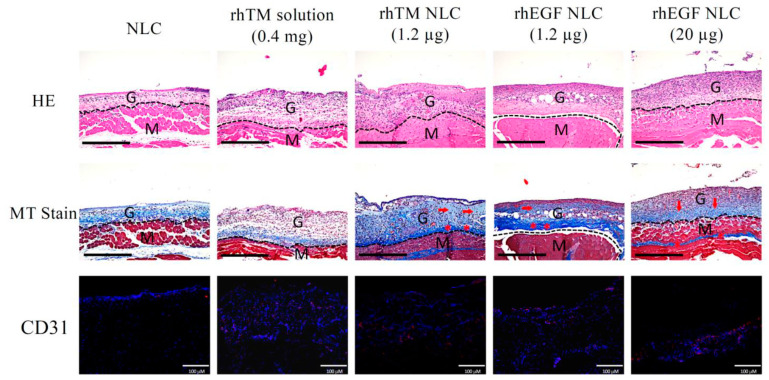
RhTM NLC 1.2 μg increased granulation tissues and enhanced collagen deposition and angiogenesis at the wound site. Wounded skins on Day 10 were collected, fixed, and embedded in paraffin. Samples were stained with hematoxylin and eosin (HE), Masson’s trichrome staining (MT), or CD31, respectively. Representative images of central area of wound were shown. In MT staining, arrows pinpointed newly formed collagen (faint blue) and asterisks denoted deposited collagen (deep blue). Dashed lines denoted boundaries between the granulation tissue and dermis/muscle. G, granulation tissue; M, muscle. In CD31 immunostaining, CD31 and nuclei were visualized as red and blue dots, respectively. Scale bar of HE and MT = 400 μm; scale bar of CD31 = 100 μm.

Wound areas of mice at day 10 were collected and analyzed by histological examination. H&E staining revealed a thin granulation tissue in groups of NLC, rhTM solution 0.4 μg, and rhEGF NLC 1.2 μg (Figure 3). In contrast, thick granulation tissues and increased re-epithelialization were observed in rhTM NLC 1.2 μg and rhEGF NLC 20 μg. MT stain showed that few newly formed collagen (faint blue) in the granulation tissue of NLC or rhTM solution 0.4 μg-treated groups. Although rhEGF NLC 1.2 μg increased collagen fibers formation and collagen deposition (blue), rhTM NLC 1.2 μg and rhEGF NLC 20 μg treatments more effectively enhanced new collagen formation and collagen deposition in the granulation tissue than NLC, rhTM solution 0.4 μg, and rhEGF NLC 1.2 μg. Besides granulation tissue and collagen formation, angiogenesis is a crucial step in wound healing process. Since CD31 is highly expressed on endothelial cells, further angiogenesis effects (neovascularization) of different formulations were analyzed by immunostaining of CD31. RhTM NLC 1.2 μg, rhEGF NLC 1.2 μg, and rhEGF NLC 20 μg obviously induced angiogenesis in the granulation tissue than NLC and rhTM solution 0.4 μg. Moreover, rhTM NLC 1.2 μg and rhEGF NLC 20 μg had more pronounced angiogenic effect than rhEGF NLC 1.2 μg. Conclusively, these results suggested that rhTM has higher potency on wound healing than rhEGF, and low-dose rhTM-NLC (1.2 μg) achieved compatible effect with high-dose rhEGF NLC 20 μg, which was demonstrated previously to improve healing in mouse and porcine wound models [18,19]. 

### 3.3. Characterization of RhTM NLC-Gel

**Figure 4:**
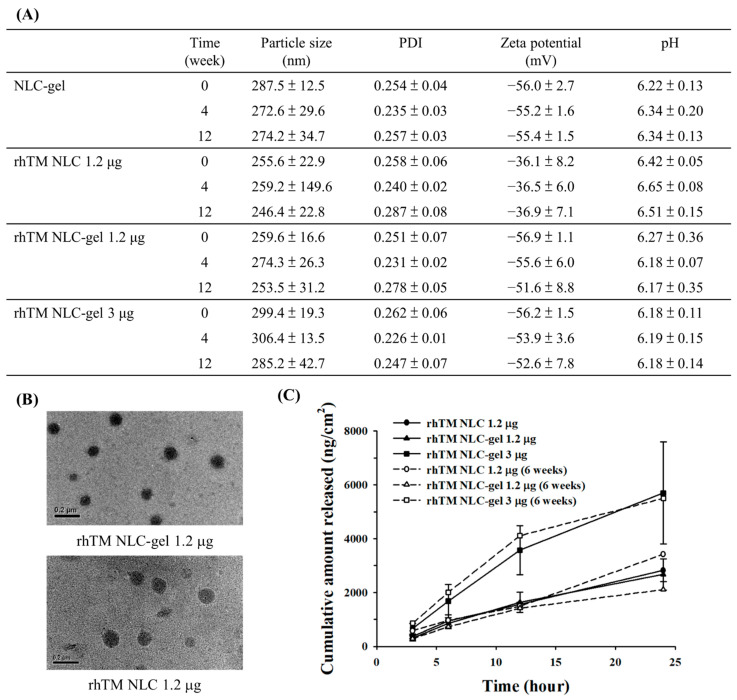
Characteristics and drug release profile of rhTM NLC-gel. (**A**) Physical characteristics of freshly prepared rhTM NLC formulations with and without gel, and after storage at 4 °C for up to 12 weeks. (*n* = 3). (**B**) Morphology of rhTM NLC and rhTM NLC-gel particles under TEM. (Scale bar = 0.2 µm). Data presented as mean ± SD. (**C**) In vitro drug release profile of freshly prepared (*n* = 4 each) rhTM NLC and rhTM NLC-gel and after storage at 4 °C for 6 weeks (*n* = 2 each). Drug release followed dose and time dependent manner.

Figure 4A depicted physical characteristics of NLC-gel and rhTM NLC-gel up to 12 weeks after preparation. Particle size slightly increased, and zeta potential became more negative after the addition of carbopol gel to rhTM NLC. When the amount of rhTM was increased to 3 µg, the particles were slightly enlarged up to around 300 nm. TEM revealed very similar particle size and morphology of both formulations (Figure 4B). The pH value of the formulation was slightly decreased after the addition of carbopol gel. No significant difference was observed in physical characteristics after 4 and 12 weeks of storage, demonstrating good physical stability of all the formulations (Figure 4A). 

Shown in Figure 4C were the in vitro drug release profiles of rhTM NLC and rhTM NLC-gel. All the formulations demonstrated constant drug release in 24 h. RhTM release profile was similar with and without addition of carbopol gel to rhTM NLC 1.2 µg, indicating that low concentration of carbopol gel does not hinder rhTM release from NLC. After 6 weeks of storage, both 1.2 µg and 3 µg rhTM NLCs maintained similar release profile as fresh prepared formulations. There was a dose-dependency of rhTM release from NLC-gel.

### 3.4. RhTM NLC-Gel 1.2 μg Is Compatible with RhTM-NLC 1.2 μg on Wound Healing

**Figure 5:**
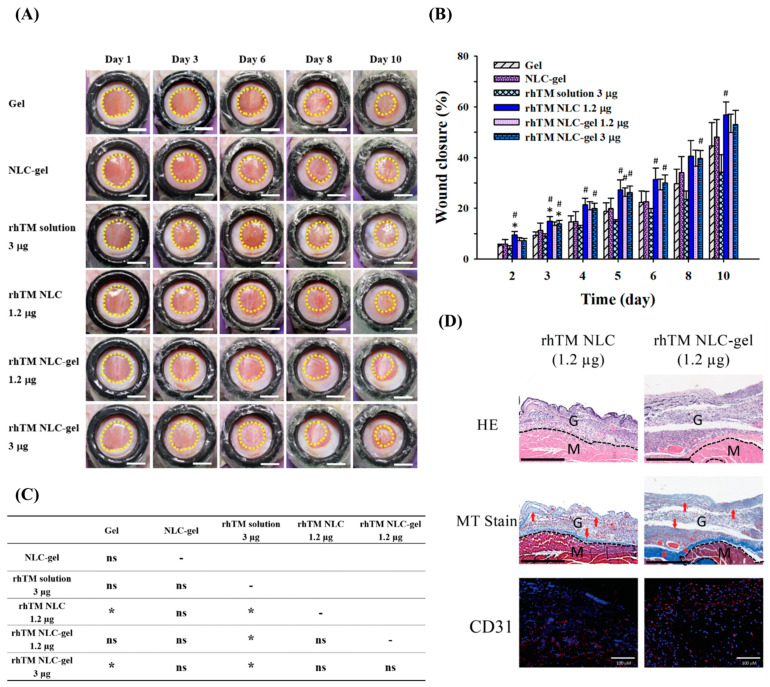
Wound healing effect of RhTM NLC-gel. (**A**) Representative wound area of streptozotocin-induced diabetic mice at Day 1 to 10 with different treatments. (*n* = 5–10 for each group; scale bar = 5 mm) (**B**) Wound closure of diabetic mice by quantification wound area in (A) and analysis following mathematical formula described in Methods. Data presented as mean ± SEM. The significance was analyzed using Student’s *t* test. *p* < 0.05 compared with gel group (*) or rhTM solution 3 µg group (#). (**C**) Multiple comparison analysis of wound closure effect between different treatment groups based on general linear model. Bonferroni post hoc test was applied. (*: *p* < 0.05; ns: *p* > 0.05) (**D**) Wounded skins on Day 10 were collected, fixed, and embedded in paraffin. The samples were stained with hematoxylin and eosin (HE), Masson’s trichrome staining (MT), or CD31, respectively. Representative images of central area of wound were shown. In MT staining, arrows pinpointed newly formed collagen (faint blue) and asterisks denoted deposited collagen (deep blue). Dashed lines denoted boundaries between the granulation tissue and dermis/muscle. G, granulation tissue; M, muscle. In CD31 immunostaining, CD31 and nuclei were visualized as red and blue dots, respectively. Scale bar of HE and MT = 400 μm; scale bar of CD31 = 100 μm.

A further in vivo animal model of wound healing was performed to evaluate the effect of 1.2 µg rhTM-NLC with or without gel, and the results were shown in Figure 5A,B. At Day 10, all three formulated rhTM, as rhTM NLC 1.2 μg (56.8 ± 5.2%), rhTM NLC-gel 1.2 μg (49.9 ± 7.2%) and rhTM NLC-gel 3 μg (53.2 ± 5.5%), had better wound healing rate than gel group (44.6 ± 9.2%) and rhTM solution 3 μg (34.2 ± 6.9%), while significance (*p* < 0.05) was only shown between rhTM NLC 1.2 μg and rhTM solution 3 μg (Figure 5B). Figure 5C summarized the significances from multiple comparison analysis of wound healing effects between all study groups. The healing effect of rhTM NLC 1.2 μg and rhTM NLC-gel 3 μg achieved significant improvement in comparison with gel group (Figure 5C). Interestingly, there was no significant difference of wound closure rate among rhTM NLC 1.2 μg, rhTM NLC-gel 1.2 μg, and rhTM NLC-gel 3 μg. Further histological examination revealed that both rhTM NLC 1.2 μg and rhTM NLC-gel 1.2 μg increased granulation tissues and re-epithelialization, and induced angiogenesis in the granulation tissue (Figure 5D). In addition, rhTM NLC-gel 1.2 μg enhanced more collagen fibers formation and collagen deposition than rhTM-NLC 1.2 μg. Taken together, both rhTM-NLC 1.2 μg and rhTM-NLC-gel 1.2 μg achieved similar beneficial effect on wound healing. Nevertheless, the NLC-gel dressing of rhTM (rhTM NLC-gel 1.2 μg) has both the advantages of sustained-release and easy adherence to wound bed that may promote its clinical application. 

### 3.5. Plasma Concentration of RhTM Post Administration of Different Formulations

Plasma concentrations of rhTM were monitored at 30 minutes, 4 h or 24 h post treatment (Table 1). Thirty minutes after the administration of rhTM solution 3 µg, a burst in rhTM concentration was found (5.6 ng/mL). Plasma rhTM concentrations post 4 h administration of rhTM NLC 1.2 µg, rhTM NLC-gel 1.2 µg and rhTM NLC-gel 3 µg administration were 0.9 ng/mL, 1.1 ng/mL and 1.9 ng/mL, respectively. A dose-dependent plasma level between rhTM NLC-gel 1.2 µg and 3 µg at 4 h was clearly indicated. While plasma concentrations post 24 h administration of rhTM solution 3 µg and rhTM NLC 1.2 µg were below limit of detection (LOD, 0.78 ng/mL), it was still detectable around 0.8 and 0.9 ng/mL for rhTM NLC-gel 1.2 µg and 3 µg administrations, respectively. These results further demonstrated sustained release of rhTM from NLC-gel in vivo, and lower plasma rhTM level was maintained for a prolonged period.

### 3.6. RhTM NLC-Gel Accelerate Keratinocyte Migration More than RhTM NLC

**Figure 6:**
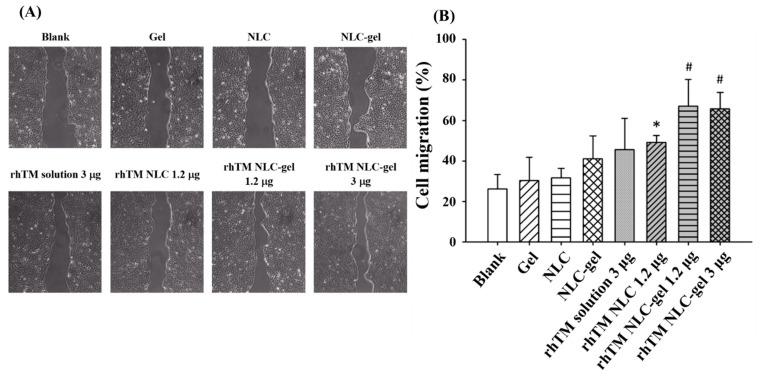
RhTM NLC-gel 1.2 μg enhanced cell migration of human keratinocyte HaCaT cells. The cells were seeded, treated with different formulations, and then photographed using OPTIKA camera and Leica microscopy system. (**A**) Representative images were shown. (**B**) The ratio of wound recovery was quantitatively determined with ImageJ software. Data are presented as mean ± SD (*n* = 4 each treatment). The significance was analyzed using Student’s *t*-test. *p* < 0.05 compared with Blank/Gel/NLC group (*) or Blank/Gel/NLC/rhTM NLC 1.2 μg group (#).

Besides increased granulation tissue, collagen formation, and angiogenesis, keratinocyte migration is another essential factor that contributes to the rate of wound healing. Therefore, further experiments on the in vitro wound healing assay of human keratinocyte HaCaT cells were performed to validate the effect of rhTM NLC and different dressings on cell migration. Figure 6A,B showed that Gel (30.4 ± 11.3%), NLC (31.7 ± 4.8%), and NLC-gel (41.1 ± 11.2%) had beneficial effects on cell migration compared to blank (26.2 ± 7.1%), indicating that these dressing may also promote cell migration. These findings may not be unexpected since previous studies have demonstrated that carbopol improved tissue perfusion and decreased the area of necrotic tissue in burn wounds [29]. RhTM solution 3 μg (45.6 ± 15.4%) also effectively induced cell migration compared to blank, but its effect was similar to NLC-gel (*p* = 0.06). However, the result was not compatible with in vivo wound healing data. Considering the different settings between in vitro and in vivo, we suggested that rhTM solution 3 μg was easily dilutable and removable from wound area by body fluid and protease in vivo but was sustained in the medium in vitro, which led to different magnitude of effects (Figure 5B and Figure 6B) [30]. On the other hand, the effect of carbopol may be magnified due to the dilution effect in the medium that resulted in lower viscosity in comparison with in vivo scenario. The three NLC formulations containing rhTM, including rhTM NLC 1.2 μg (49.1 ± 3.5%), rhTM NLC-gel 1.2 μg (67.1 ± 13.3%) and rhTM NLC-gel 3 μg (65.7 ± 8.3%), significantly enhanced cell migration compared to blank, Gel, or NLC group after 48 h treatment. Gel formulated rhTM NLC-gel 1.2 μg and rhTM NLC-gel 3 μg also stimulated cell migration more than the no-gel rhTM NLC 1.2 μg. These data imply that carbopol in gel formulations may have synergistic effect with rhTM and NLC on cell migration. In addition, the data pinpointed that low-dose rhTM NLC-gel 1.2 μg achieved similar effect with high-dose rhTM-NLC-gel 3 μg. Taken together, the NLC-gel dressing itself not only elicited healing effect, but also sustained the residence of rhTM on the wound area and protected rhTM activity away from protease degradation. 

In summary, the first set of wound healing experiments demonstrated that rhTM NLC 1.2 μg achieved best wound closure among all the treatment groups, and the second set of experiments substantiated that incorporation of carbopol with rhTM NLC did not affect its efficacy. Increasing dose of rhTM in NLC-gel from 1.2 μg to 3 μg did not further benefit would healing. It is known that, although a viscous vehicle like carbopol gel may prolong residence of formulation on the wound, it is also possible to slowdown rhTM release from NLC-gel. It might be because that rhTM is the main factor promoting wound healing, and the effect from low concentration of carbopol may not be enough to be detected in vivo. Nevertheless, rhTM NLC-gel did show significant acceleration in cell migration when compared with all other groups including rhTM NLC (Figure 6). All together, these data support that NLC-gel formulation of rhTM can be as beneficial for wound healing treatment as rhTM in NLC only. 

## 4. Conclusions

The importance of using growth factors on wound healing is rapidly increasing; however, having high concentration of these growth factors entering the circulation may cause concerns such as risk of cancer. Both treatments with rhTM NLC 1.2 μg and rhTM NLC-gel 1.2 μg increased granulation tissues and re-epithelialization, and induced angiogenesis in the granulation tissue, leading to improvement of chronic wound healing. In addition, when compared with solution form, plasma concentrations of rhTM post applications of NLC and NLC-gel formulations were lower and more sustained in 24 h, which likely resulting in longer treatment time. The developed rhTM NLC and rhTM NLC-gel formulations are stable, easy to prepare, convenient to apply to the wound with reduced systemic exposure, which may warrant potential delivery systems for the care of chronic wound patients. The gel form with slight viscosity may be more advantageous for topical application.

## Figures and Tables

**Table 1:** Plasma rhTM levels post different treatments.

	Plasma Concentration (ng/mL)		
Time (after Administration)	30 min	4 h	24 h
NLC-gel		<LOD	<LOD
rhTM solution 3 μg	5.4/5.8		<LOD
rhTM NLC 1.2 μg		0.9 ± 0.0	<LOD
rhTM NLC-gel 1.2 μg		1.1 ± 0.2	0.8 ± 0.3
rhTM NLC-gel 3 μg		1.9 ± 0.2	0.9 ± 0.5

Data are presented as mean±SD. N = 3–4, except n = 2 for rhTM solution 3 μg.

## Data Availability

The data presented in this study are available on request from the corresponding author.

## Supplementary Materials

The following are available online at https://www.mdpi.com/article/10.3390/pharmaceutics13091386/s1, Figure S1: Characteristics of NLC formulations in 3D bar graph, Figure S2: RhTM release profile from NLC formulations, Figure S3: Wound closure % of rhTM NLC 1.2 μg after 1 year of storage at 4 °C versus freshly prepared. Table S1: Characteristics of NLC formulations, Table S2: Characteristics of NLC formulations, Table S3: Encapsulation efficiency of rhTM in NLC.
