# Supplementary material for: Nanostructured Lipid Carrier Gel Formulation of Recombinant Human Thrombomodulin Improve Diabetic Wound Healing by Topical Administration
Source: Pharmaceutics. 2021 Sep 2;13(9):1386. doi: 10.3390/pharmaceutics13091386 (PMC8469737; doi:10.3390/pharmaceutics13091386)
Supplement: Supplementary file 1 [file pharmaceutics-13-01386-s001.zip › pharmaceutics-1336237-supplementary.pdf]

Supplementary figures and tables

Table S1. Characteristics of NLC formulations

Solid lipid / Oil	Solid lipid：oil	Particle size (nm)	PDI	Zeta potential (mV)	Sample size
ATO5/Miglyol	8：2	360.4 ± 98.2	0.578 ± 0.31	-39.2 ± 4.4	3
ATO5/Miglyol	7：3	233.0 ± 38.1	0.309 ± 0.12	-39.2 ± 3.6	3
ATO5/Miglyol	6：4	224.0 ± 20.0	0.292 ± 0.09	-39.5 ± 0.9	3
ATO5/Miglyol	5：5	223.6 ± 17.9	0.268 ± 0.05	-41.0 ± 5.8	11
ATO5/Miglyol	4：6	357.2 ± 85.7	0.378 ± 0.06	-45.2 ± 4.4	3
ATO5/Captex	8：2	188.2 ± 5.2	0.231 ± 0.01	-39.9 ± 0.9	3
ATO5/Captex	7：3	186.9 ± 0.8	0.229 ± 0.01	-40.2 ± 1.2	3
ATO5/Captex	6：4	199.0 ± 10.4	0.247 ± 0.01	-41.7 ± 1.7	3
ATO5/Captex	5：5	237.7 ± 27.3	0.314 ± 0.05	-38.6 ± 5.8	6
ATO5/Captex	4：6	292.5 ± 7.1	0.332 ± 0.04	-44.1 ± 2.4	3
Geleol/Miglyol	8：2	407.9 ± 59.1	0.720 ± 0.01	-29.1 ± 4.2	3
Geleol/Miglyol	7：3	299.6 ± 74.2	0.583 ± 0.18	-30.4 ± 1.8	3
Geleol/Miglyol	6：4	224.5 ± 31.5	0.486 ± 0.07	-30.4 ± 1.9	3
Geleol/Miglyol	5：5	154.2 ± 23.6	0.308 ± 0.02	-34.3 ± 3.5	5
Geleol/Miglyol	4：6	148.7 ± 6.6	0.267 ± 0.01	-34.1 ± 1.4	3
Geleol/Captex	8：2	295.1 ± 127.5	0.548 ± 0.29	-24.7 ± 2.1	3
Geleol/Captex	7：3	316.5 ± 20.2	0.606 ± 0.03	-25.7 ± 2.0	3
Geleol/Captex	6：4	241.9 ± 24.9	0.484 ± 0.04	-26.3 ± 1.5	3
Geleol/Captex	5：5	155.8 ± 11.4	0.235 ± 0.01	-29.4 ± 3.2	5
Geleol/Captex	4：6	163.3 ± 8.5	0.207 ± 0.01	-35.9 ± 4.4	3

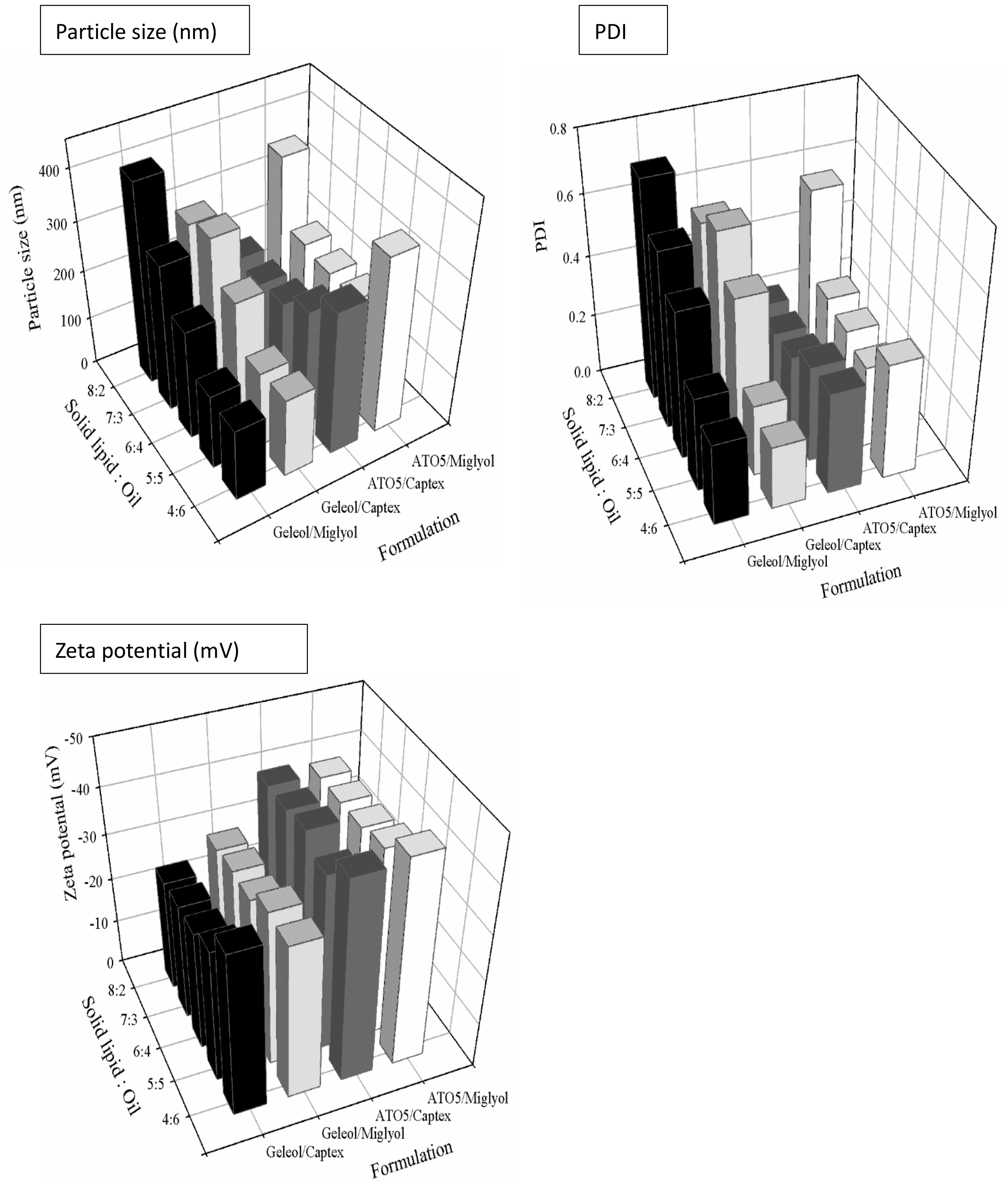

Figure S1. Characteristics of NLC formulations in 3D bar graph.

Table S2. Characteristics of NLC formulations

Drug content (μg)	Solid lipid / Oil	Solid lipid：oil	Particle size (nm)	PDI	Zeta potential (mV)	Sample size
--	ATO5/Miglyol	5：5	223.6 ± 17.9	0.268 ± 0.05	-41.0 ± 5.8	11
120	ATO5/Miglyol	5：5	220.2 ± 12.3	0.238 ± 0.04	-43.1 ± 0.9	3
--	ATO5/Captex	5：5	237.7 ± 27.3	0.314 ± 0.05	-38.6 ± 5.3	6
120	ATO5/Captex	5：5	182.3	0.230	-37.1	2
--	Geleol/Miglyol	5：5	154.2 ± 23.6	0.308 ± 0.02	-34.3 ± 3.5	5
120	Geleol/Miglyol	5：5	144.9	0.314	-27.2	2
--	Geleol /Captex	5：5	155.8 ± 11.4	0.235 ± 0.01	-29.4 ± 3.2	5
120	Geleol /Captex	5：5	163.3	0.249	-32.4	2

Table S3. Encapsulation efficiency of rhTM in NLC

Formulation	Free form rhTM (%)	Encapsulation efficiency (%)
AM	6.5 ± 1.0	93.5 ± 1.0
AC	4.8 ± 1.8	95.2 ± 1.8
GM	7.9 ± 4.8	92.1 ± 4.8
GC	6.5 ± 2.3	93.5 ± 2.3

A: ATO5

G: Geleol

M: Miglyol

C: Captex

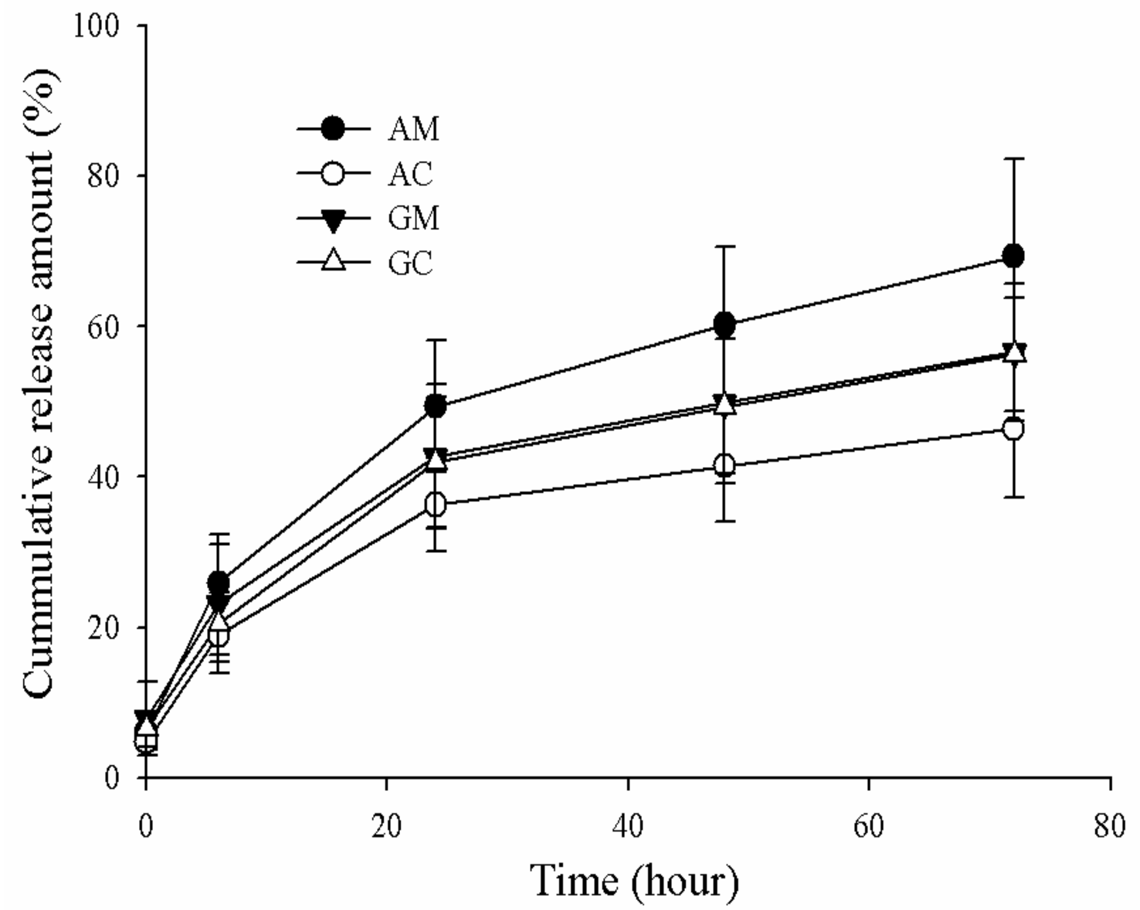

Figure S2. RhTM release profile from NLC formulations. Highest release of rhTM from AM-NLC among the 4 formulations

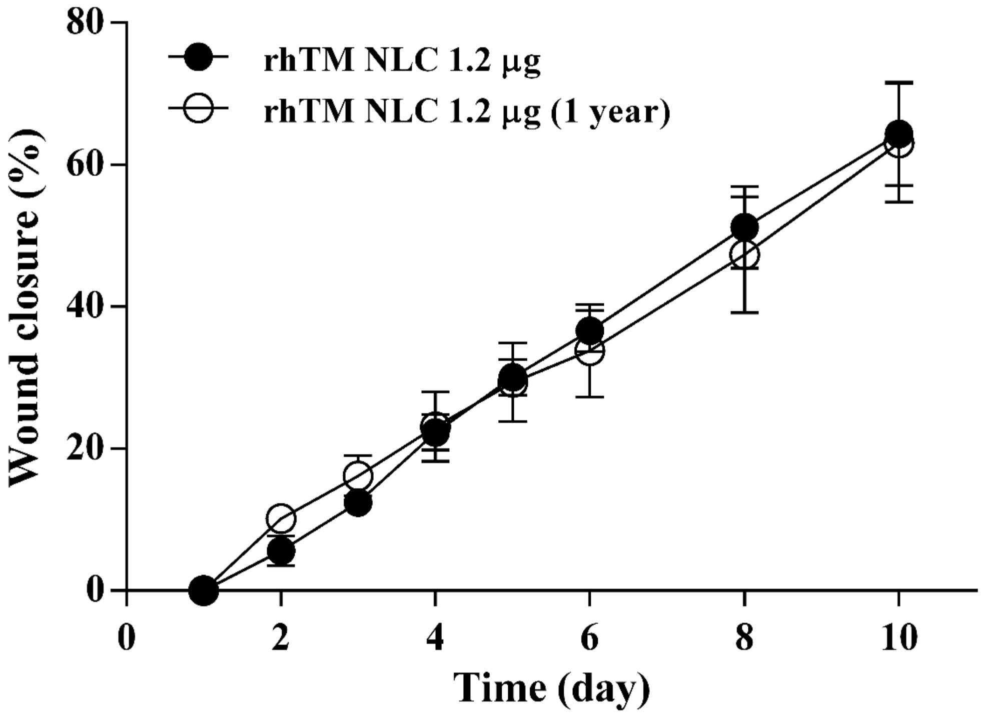

Figure S3. Wound closure % of rhTM NLC 1.2 μg after 1 year of storage at 4℃ versus freshly prepared.
